# Quantifying compositional variability in microbial communities with FAVA

**DOI:** 10.1073/pnas.2413211122

**Published:** 2025-03-10

**Authors:** Maike L. Morrison, Katherine S. Xue, Noah A. Rosenberg

**Affiliations:** ^a^Department of Biology, Stanford University, Stanford, CA 94305

**Keywords:** compositional variability, FST, microbial communities, microbiomes, population genetics

## Abstract

Microbial communities vary across space, time, and individual hosts, generating a need for statistical methods capable of quantifying variability across multiple microbiome samples at once. To understand heterogeneity across microbiome samples from different host individuals, sampling times, spatial locations, or experimental replicates, we present FAVA (*F*_*ST*_-based Assessment of Variability across vectors of relative Abundances), a framework for characterizing compositional variability across two or more microbiome samples. FAVA quantifies variability across many samples of taxonomic or functional relative abundances in a single index ranging between 0 and 1, equaling 0 when all samples are identical and 1 when each sample is entirely composed of a single taxon (and at least two distinct taxa are present across samples). Its definition relies on the population-genetic statistic *F*_*ST*_, with samples playing the role of “populations” and taxa playing the role of “alleles.” Its mathematical properties allow users to compare datasets with different numbers of samples and taxonomic categories. We introduce extensions that incorporate phylogenetic similarity among taxa and spatial or temporal distances between samples. We demonstrate FAVA in two examples. First, we use FAVA to measure how the taxonomic and functional variability of gastrointestinal microbiomes across individuals from seven ruminant species changes along the gastrointestinal tract. Second, we use FAVA to quantify the increase in temporal variability of gut microbiomes in healthy humans following an antibiotic course and to measure the duration of the antibiotic’s influence on temporal microbiome variability. We have implemented this tool in an R package, *FAVA*, for use in pipelines for the analysis of microbial relative abundances.

## Significance

Studies of microbial community composition across time, space, or biological replicates often rely on summary statistics that analyze just one or two samples at a time. Although these statistics effectively summarize the diversity of one sample or the compositional dissimilarity between two samples, they are ill-suited for measuring variability across many samples at once. Measuring compositional variability among many samples is key to understanding the temporal stability of a community across multiple time points or the heterogeneity of microbiome composition across multiple experimental replicates or host individuals. Our proposed framework, *F*_*ST*_-based Assessment of Variability across vectors of relative Abundances (FAVA), meets the need for a statistic summarizing compositional variability across many microbiome samples all at once.

Understanding the compositional variability of microbial communities across space, time, or host individuals is important for characterizing these communities and their relationships with biological variables of interest (1–13). For example, studies of microbiome composition have found that microbiome compositions are often more variable across dysbiotic individuals than across healthy individuals (14), the microbial communities of infants tend to be more variable across individuals than those of adults (15), and gut and tongue microbiomes that are more diverse may be less temporally variable (5). Despite its biological importance, however, compositional variability is difficult to directly quantify with existing methods.

**Fig. 1.**
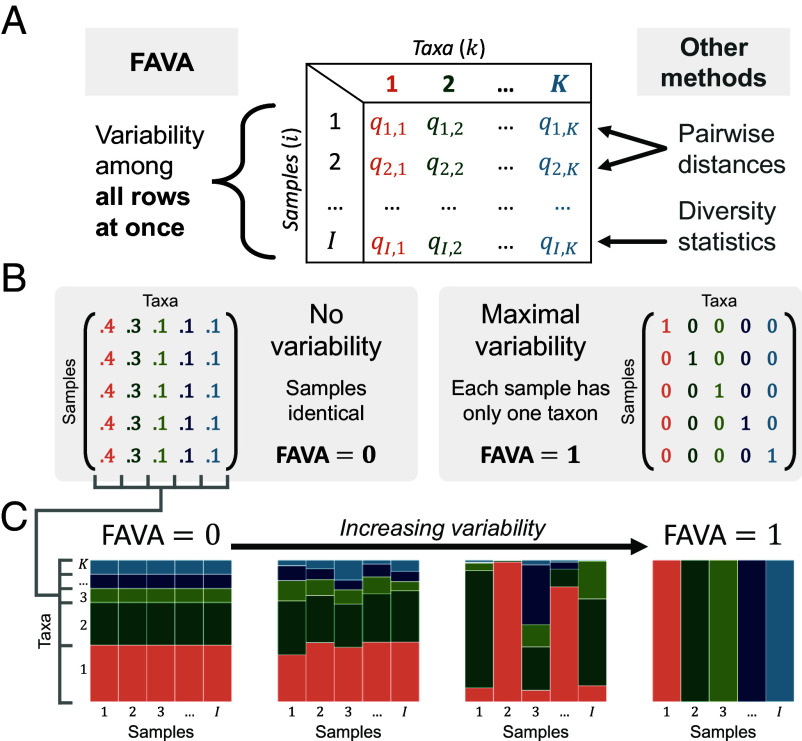
FAVA quantifies compositional variability across many abundance vectors in a single number. (*A*) FAVA is computed among the rows of a matrix, with rows representing microbiome samples, columns corresponding to microbial taxa such as OTUs or families, and entries representing relative abundances. FAVA quantifies variability across many rows in a single number, distinguishing it from other methods, which analyze just one or two rows at a time. (*B*) Given the number of samples (*I*) and number of taxa (*K*), variability is minimized if the rows are identical, corresponding to the case where each sample is the same as every other sample. Variability is maximized if each sample contains just one taxon, as long as there are at least two different taxa present across all samples. When plotted as a relative abundance plot (e.g., panel *C*), each row of these matrices is visualized as a vertical bar. The matrix pivots 90 degrees when visualized. (*C*) For the four matrices, variability across samples increases as samples become less similar; from *Left* to *Right*, the values of FAVA for the matrices are 0, 0.006, 0.452, and 1.

We define “compositional variability” as variability across two or more compositional vectors—lists of proportions that sum to 1 (Fig. 1*A*). Compositional variability is minimized when the vectors have identical compositions; it is maximized when each vector contains a single category at 100% frequency and at least two categories have nonzero frequency in the sum of the vectors (Fig. 1 *B* and *C*). We focus on vectors that represent the composition of microbiome samples. These vectors’ entries represent relative abundances of taxonomic categories such as operational taxonomic units (OTUs), species, or even functional categories such as gene classifications (16–18). Each vector can represent the composition of a microbiome sample from a distinct timepoint, spatial location, host individual, or replicate. Compositional variability can therefore represent temporal stability, spatial heterogeneity, interhost diversity, or repeatability (3, 9, 12, 18–24).

Traditionally, microbiome studies have used statistics such as the Shannon and Gini-Simpson indices (25), the Jensen–Shannon divergence (26), and the Bray–Curtis dissimilarity (27). Single-sample diversity statistics such as the Shannon and Gini-Simpson indices quantify the variability of microbiome samples considered individually, answering questions such as “Which of these microbiomes is the most diverse?” Pairwise statistics, such as the Jensen–Shannon divergence, Jaccard index, and Bray–Curtis dissimilarity compare the compositions of two samples, answering questions such as “How does the composition of a perturbed microbial community compare to a preperturbation reference sample?” Although these tools are valuable when variability is of interest in one sample or between two samples, they are less well suited to scenarios in which three or more samples are of interest, as they only consider one or two samples at once.

Studies that seek to quantify variability across many samples are often limited to computing summary measures of each sample, such as diversity indices (5, 18), principal component coefficients (14, 28), or the abundances of individual taxa (10, 29), and computing the variability across samples of these summary statistics. However, this approach measures the variability of a summary statistic, not the variability of the microbiome composition itself. Because it is possible for very different compositions to produce similar values of a summary statistic, such indirect variability measures potentially obscure large differences among samples.

Consider for illustration the study of Flores et al. (5), which aimed to compare regions of the body in terms of their temporal variability in microbiome composition. For 85 adults, they profiled the microbiomes of four body habitats weekly for three months. They measured temporal variability by computing diversity statistics such as the Shannon index for each temporal sample, then computing the coefficient of variation of the Shannon index over time for each of the 85 individuals and four body regions. This approach quantifies the variability of the Shannon diversity, not the variability of the microbiome composition itself. Because equal values of the Shannon index can be obtained for two communities with similar compositions, as well as for two communities with quite different compositions, this method could assign time series with dramatically different levels of compositional change the same coefficient of variation, obscuring meaningful differences among them.

Here, we present FAVA, a statistic that quantifies variability of microbiome composition across many microbiome samples. In a single number, FAVA measures variability of microbial composition across arbitrarily many microbiome samples, summarizing large datasets. The measure allows for the optional inclusion of similarities among taxonomic categories (e.g., phylogenetic similarity) and for optional nonuniform weighting of samples (e.g., to account for uneven sampling time intervals). FAVA, which stands for an *F*_*ST*_-based Assessment of Variability across vectors of relative Abundances, is based on the statistic *F*_*ST*_, which originated in population genetics to quantify variability across vectors of allele frequencies for multiple populations. FAVA takes values between 0 and 1, equaling 0 when all sampled microbiome compositions are identical and 1 when each sample contains only a single taxon and at least two distinct taxa are present across samples (Fig. 1 *B* and *C*). It has mathematical properties that allow it to be used to compare variability among sets of samples with very different numbers of taxa or datasets with very different numbers of samples.

We demonstrate the FAVA framework with two datasets, one containing spatial samples along the gastrointestinal tract of seven species of ruminants, and the other describing time series of gut microbiome samples from 22 human individuals who experienced an antibiotic perturbation. In the ruminant dataset, we identify substantially higher interindividual variability in the stomach and small intestine than in the large intestine, supporting the view that substantial microbiome variability is obscured when gastrointestinal communities are sampled through fecal samples alone (18). In the human dataset, we show that temporal variability in microbiome composition is elevated following an antibiotic perturbation, and that just half of subjects return to low levels of temporal variability in the 30 d following completion of the antibiotic.

## Results

### Definition of FAVA.

The composition of a microbial community is most commonly described in terms of relative abundances of OTUs, species, bacterial families, or other units, including functional units such as gene categories. Matrices of such abundances are central to software widely used for the analysis of microbiome data, such as *Phyloseq* (30) and *QIIME2* (31). In an “OTU table,” denoted *Q*, each row represents a microbial community sample, each column represents a distinct taxon, and the entry $q_{i,k}$ represents the relative abundance of taxon *k* in sample *i* (Fig. 1*A*). The samples in an OTU table represent samples of microbial communities that could vary in their sampling location, sampling time, and subject or replicate. Throughout this paper, we use “sample *i*” to refer to row *i* of the OTU table.

FAVA quantifies variability across the rows of an OTU table (Fig. 1*A*). If the rows represent samples from different time points for one subject, FAVA is a measure of the temporal stability of the community. If the rows represent different sampling locations, FAVA quantifies the spatial heterogeneity of the community. FAVA can be independently computed on disjoint subsets of the rows of an OTU table. For example, to measure the temporal variability in microbiome composition for each of many subjects, the entire matrix would contain many subjects and time points, and matrix subsets containing just one subject and many time points could be separately analyzed. The measure ranges between 0 (no variability) and 1 (maximal variability) and can be used to compare the variabilities of multiple sets of samples (Fig. 1 *B* and *C*).

FAVA is based on the population-genetic statistic *F*_*ST*_, which is used mainly to measure variability of allele frequencies across populations but can also apply for other types of compositional data (32–34). We apply FAVA to microbiomes by analyzing microbial taxon abundances in place of allele frequencies, and microbiome samples in place of populations.

*F*_*ST*_ is defined in terms of the population-genetic statistic heterozygosity, mathematically equivalent to the Gini-Simpson diversity in ecology. For a sample *i* with k=1,2,…,K taxa with abundances $q_{i,k}$, the Gini-Simpson diversity of the sample is the probability that two random draws from the sample do not belong to the same taxon (25):

[1] $$\Delta \left(q_{i,1},q_{i,2},\ldots ,q_{i,K}\right)=1-\sum_{k=1}^{K}{\left(q_{i,k}\right)}^{2}.$$

$\Delta (q_{i,1},q_{i,2},\ldots ,q_{i,K})=0$ if and only if some taxon has abundance 1 and all others have abundance 0 (i.e., $q_{i,k^{\prime }}=1$ for some $k^{\prime }$, and $q_{i,k}=0$ for all $k\neq k^{\prime }$). $\Delta \left(q_{i,1},q_{i,2},\ldots ,q_{i,K}\right)=1-\frac{1}{K}$, its maximum given *K*, if and only if all taxa are equally abundant (i.e., $q_{i,k}=\frac{1}{K}$ for all k=1,2,…,K).

*F*_*ST*_ proceeds by computing this diversity index on the set of all i=1,2,…,I microbiome samples (i.e., rows of the OTU table, *Q*) in two ways. The mean sample Gini-Simpson diversity, $\Delta_{S}$, is computed by averaging the Gini-Simpson diversities of the samples:

[2] $$\Delta_{S}\left(Q\right)=\frac{1}{I}\sum_{i=1}^{I}\Delta \left(q_{i,1},q_{i,2},\ldots ,q_{i,K}\right)=1-\frac{1}{I}\sum_{i=1}^{I}\sum_{k=1}^{K}{\left(q_{i,k}\right)}^{2}.$$

The total Gini-Simpson diversity, $\Delta_{T}$, is the Gini-Simpson index if the samples were pooled. It is computed by first calculating the centroid of the samples (the vector of mean taxon abundances over all *I* samples) and then computing the Gini-Simpson diversity of the centroid:

[3] $$\Delta_{T}\left(Q\right)=\Delta \left({\bar{q}}_{1},{\bar{q}}_{2},\ldots ,{\bar{q}}_{K}\right)=1-\sum_{k=1}^{K}{\left(\frac{1}{I}\sum_{i=1}^{I}q_{i,k}\right)}^{2},$$

where ${\bar{q}}_{k}=\frac{1}{I}\sum_{i=1}^{I}q_{i,k}$. In short, we compute $\Delta_{S}$ by first computing the Gini-Simpson index for all samples and then averaging, and we compute $\Delta_{T}$ by first averaging all samples and then computing the Gini-Simpson index.

The population-genetic statistic *F*_*ST*_ is the normalized difference between these two quantities:

[4] $$F_{\mathrm{ST}}\left(Q\right)=\frac{\Delta_{T}\left(Q\right)-\Delta_{S}\left(Q\right)}{\Delta_{T}\left(Q\right)}.$$

Assuming $\Delta_{T}\left(Q\right)>0$, *F*_*ST*_ equals 0 if and only if $\Delta_{T}\left(Q\right)=\Delta_{S}\left(Q\right)$, which occurs if and only if all *I* samples are identical (Fig. 1*B*, *Left*-hand side). *F*_*ST*_ equals 1 if and only if $\Delta_{S}\left(Q\right)=0$ and $\Delta_{T}\left(Q\right)>0$, which occurs if and only if each sample has only a single taxon, and there are at least two distinct taxa present across all samples (Fig. 1*B*, *Right*-hand side). In the language of OTU tables, *F*_*ST*_ equals 0 if and only if all rows of the OTU table are identical, and it equals 1 if and only if each row contains a single one and *K* − 1 zeroes (and at least two columns contain a one). *F*_*ST*_ can be viewed as a measure of how well mixed the samples are across a dimension of interest: If all samples are perfectly mixed, then their compositions are identical and *F*_*ST*_ is 0.

Possible values of *F*_*ST*_ range between 0 and 1 for any sample size. However, when the number of samples is small, *F*_*ST*_ can be constrained by the mean frequency of the dominant taxon, especially if this frequency is close to 0 or 1 (35). Normalizing *F*_*ST*_ by its theoretical upper bound conditional on the number of samples and the mean frequency of the most abundant taxon ($F_{\mathrm{ST}}^{\text{max}}$) can account for this property, allowing for differences in variability to be distinguished from differences in the abundance of the dominant taxon. However, because the normalized statistic is divided by a theoretical upper bound possibly less than 1, $F_{\mathrm{ST}}/F_{\mathrm{ST}}^{\text{max}}$ can equal one without satisfying the conditions described in Fig. 1*C*. The normalized statistic $F_{\mathrm{ST}}/F_{\mathrm{ST}}^{\text{max}}$ (33) is included as an option in the *FAVA* R package. Further discussion of when to consider normalizing *F*_*ST*_ by this upper bound is included in the *FAVA* R package’s vignette on microbiome data analysis.

*F*_*ST*_ has favorable mathematical properties that make it well-suited for comparisons of compositional variability among datasets with different values of the number of taxa *K*. Under a Dirichlet mathematical model for the probabilities of the relative abundances in a sample, the expected value of *F*_*ST*_ is linearly related to the Dirichlet variance and does not depend on the number of samples (*I*), the number of categories (*K*), or the category abundance parameters ( 33, equations A5 and A11). *SI Appendix*, Fig. S1*A* further demonstrates in Dirichlet simulations that FAVA is comparable between datasets with very different numbers of categories (3 and 99 taxa).

Having introduced FAVA and its mathematical properties, we now apply the method to data. Here, we focus on two example applications: using FAVA to quantify variability across individuals in the ruminant gastrointestinal tract, and using weighted FAVA to quantify temporal variability in the human gut in response to antibiotic perturbation.

### Gastrointestinal Microbiome Variability Across Ruminant Species.

Along the vertebrate gastrointestinal tract, factors such as nutrient availability, pH, and oxygen level vary substantially, shaping the types, abundances, and functions of resident microbes (18, 36, 37). Quantifying the across-host variability of microbiomes along the gastrointestinal tract can elucidate spatially structured, in vivo community assembly.

**Fig. 2.**
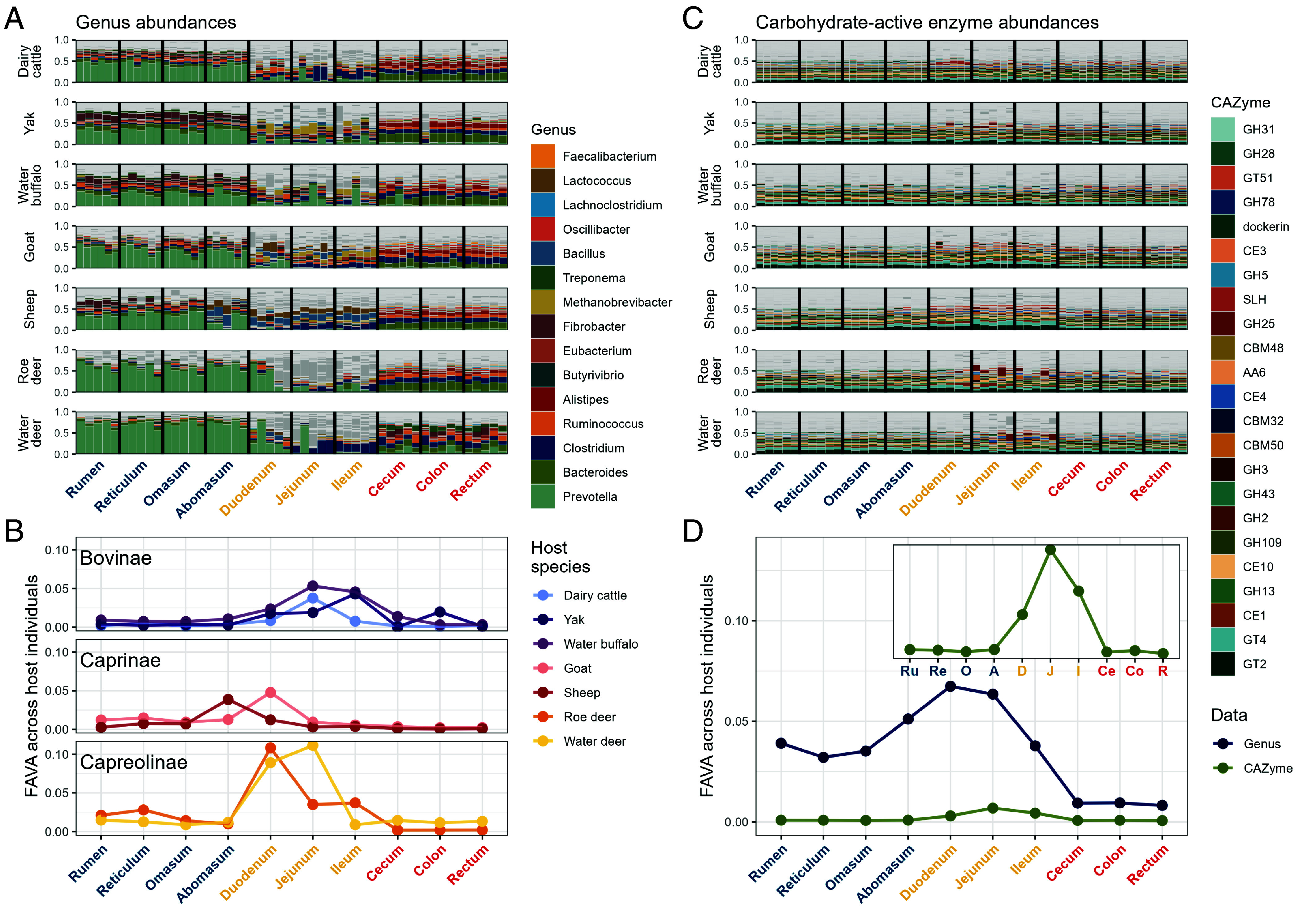
FAVA quantifies taxonomic and functional variability across host individuals and species along the ruminant gastrointestinal tract. (*A*) Relative abundances of genera across gastrointestinal regions for seven host species: dairy cattle (*n* = 6), yak (*n* = 5), water buffalo (*n* = 5), goat (*n* = 6), sheep (*n* = 5), roe deer (*n* = 5), and water deer (*n* = 5). Vertical black lines delimit the 10 gastrointestinal regions: rumen, reticulum, omasum, and abomasum for the stomach (blue); duodenum, jejunum, and ileum for the small intestine (yellow); and cecum, colon, and rectum for the large intestine (red). The ordering of these regions matches the ordering of the regions along the digestive tract. Each colored vertical bar represents the genus-level composition of a microbiome sample from one gastrointestinal region within one host individual. The horizontal ordering of individuals is consistent across regions. Of the 4,134 genera across all samples, the 15 with mean across-sample abundance greater than 1% are colored; all other genera are dark gray. Light gray horizontal lines delineate breaks between different genera. Genera are plotted in order of decreasing genus abundance across all samples and host species, from *Bottom* (most abundant) to *Top* (least abundant). (*B*) Across-individual variability of genus abundances within each gastrointestinal region for each host species. Each dot corresponds to the value of FAVA computed across all samples within a gastrointestinal region for one species. Species are grouped into panels by subfamily. (*C*) Carbohydrate-active enzyme (CAZyme) relative abundances across gastrointestinal regions for each host species. We color the 23 of 350 CAZymes that are present at more than 1% abundance across all samples. (*D*) Taxonomic (genus) versus functional (CAZyme) variability across the 37 individuals from 7 host species for each gastrointestinal region. For each gastrointestinal region, we compute FAVA using relative abundances of either genera (navy, panel *A*) or CAZYme categories (green, panel *C*). We compute variability across samples from the 37 host individuals, irrespective of host species. Each dot corresponds to the value of FAVA for a gastrointestinal region across the 37 host individuals. The *Inset* panel magnifies the plot for CAZymes.

We here use FAVA to quantify the variability of ruminant gastrointestinal microbiomes across individuals from seven host species. We analyze data from Xie et al. (38), who used shotgun metagenomics to profile samples collected along the gastrointestinal tracts of 37 individuals across seven species of ruminants (Fig. 2). For each individual, Xie et al. (38) collected samples from ten gastrointestinal regions: the rumen, reticulum, omasum, and abomasum of the stomach (Fig. 2, blue *x*-axis labels); the duodenum, jejunum, and ileum of the small intestine (Fig. 2, yellow *x*-axis labels); and the cecum, colon, and rectum of the large intestine (Fig. 2, red *x*-axis labels). Xie et al. (38) used their metagenomic sequences to infer abundances of both taxonomic categories, namely microbial genera (Fig. 2*A*), and functional categories, such as carbohydrate-active enzymes (Fig. 2*C*). We computed FAVA on these data in order to understand which gastrointestinal regions have the most and least variable genus-level compositions across individuals within each host species (Fig. 2*B*) and to compare the across-individual variability of microbial genera to the across-individual variability of functional gene categories throughout the gastrointestinal tract, across all seven host species (Fig. 2*D*).

#### Variability in genus abundances.

The genus-level compositions of the microbiome samples are shown in Fig. 2*A*. Across host species, all regions of the stomach (blue *x*-axis labels) are dominated by bacteria in the genus *Prevotella*. The samples from the small intestine (yellow *x*-axis labels), on the other hand, are much less homogeneous, with dramatic inconsistency across individuals even within a single region and host species. Samples from the large intestine (red *x*-axis labels) possess a few genera, such as *Bacteroides* (olive), *Clostridium* (navy), and *Ruminococcus* (peach), at similar frequencies across host species and regions.

We first used FAVA to quantify for each region the variability of microbial genus abundances across samples from the same host species (Fig. 2*B*). In order to do this calculation, we first partitioned the 370 samples of microbial genus abundances (37 individuals × 10 regions) into 70 matrices, each corresponding to one of the seven host species and one of the ten gastrointestinal regions. In each matrix, rows represent microbiome samples (vertical bars in Fig. 2*A*) and columns represent microbial genera. We then computed FAVA across the rows of each matrix, quantifying in a single number the variability across all 5 or 6 samples in the same host species and gastrointestinal region.

We find that FAVA is significantly higher in regions of the small intestine than in regions of the other two organs: Wilcoxon rank-sum tests comparing the 21 small-intestine FAVA values (3 small-intestine regions × 7 host species) to the 28 stomach FAVA values (4 stomach regions × 7 host species) or to the 21 large-intestine FAVA values (3 large-intestine regions× 7 host species) have one-sided *P* = 0.002 and $P<10^{-5}$, respectively. FAVA is also lower in large-intestine regions than in stomach regions (Wilcoxon rank-sum test, one-sided *P* = 0.001). These results accord with a view that monitoring microbiome composition via stool sampling alone may obscure substantial among-individual variability present upstream in the digestive tract (18, 36).

Next, we measured the compositional variability of genus abundances for each gastrointestinal region across all host species (Fig. 2*A*, vertical slices delimited by black lines). We partitioned the same 370 samples of microbial genus abundances into 10 matrices, one per gastrointestinal region. Again, matrix rows represent microbiome samples and columns represent microbial genera. We then used FAVA to quantify, for each region, the variability of genus abundances across the 37 individuals from the seven host species (Fig. 2*D*, navy). We compared FAVA values between pairs of matrices by bootstrapping. For each pair, we generated 1,000 pairs of bootstrap replicate matrices under the null hypothesis that there is no difference in variability between them; in particular, we generated each bootstrap-resampled matrix by drawing rows from both matrices with replacement. We next computed the difference in FAVA values between resampled pairs of matrices in order to generate a null distribution of the difference in FAVA values between the matrices. Comparing the observed difference in FAVA values between the original two matrices to this distribution yields a *P*-value for the null hypothesis that the observed difference in FAVA values is 0; further details appear in *Materials and Methods*.

We find that FAVA values are highest at the distal end of the stomach and proximal end of the small intestine (pairwise bootstrap comparisons between duodenum and all regions except for abomasum and jejunum, one-sided *P* < 0.04 for all 7 pairs). FAVA values are lowest in the large intestine (21 pairwise bootstrap comparisons between each of the three large-intestine regions and the seven other regions: one-sided *P* < 0.001 for 9 comparisons to small-intestine regions; one-sided *P* < 0.01 for 3 comparisons to the abomasum; one-sided *P* < 0.06 for 6 comparisons to the reticulum or omasum; and one-sided *P* < 0.04 for 3 comparisons to the rumen). Variability changes continuously along the gastrointestinal tract, in the sense that the FAVA value for each region is generally between or near those of its preceding and subsequent regions.

#### Variability in microbiome function.

The functional profile of a microbial community, measured in terms of the types of genes present, provides information not captured by the community’s taxonomic composition (39). Abundances of gene functional categories are generated by mapping shotgun metagenomic reads to a database of gene sequences grouped by function. We focus here on 350 carbohydrate-active enzymes (CAZymes), which determine the ability of a microbial community to break down complex carbohydrates (40) (Fig. 2*C*). In general, we expect to see lower variability in functional categories such as CAZymes than in taxonomic categories such as genera because of the phenomenon of functional redundancy: multiple microbial taxa carry out similar metabolic processes, allowing the taxonomic composition of a community to vary without influencing its function (1, 12, 16, 39, 41, 42). Because FAVA can be computed irrespective of the number of categories, we can use it to compare the variabilities of very different types of data, such as taxonomic and functional abundances.

To quantify functional redundancy in each region of the gastrointestinal tract, we compared the variability of genus abundances across the 37 host individuals to the variability of CAZyme abundances across the same 37 host individuals. We expect functional redundancy to keep CAZyme variability lower than genus variability, with a larger difference between the taxonomic and functional variability indicating stronger functional redundancy. We quantified genus-level taxonomic variability in each of 10 gastrointestinal regions by computing FAVA across vectors of genus abundances sampled from the 37 host individuals, irrespective of host species. This computation resulted in 10 values of FAVA, one per gastrointestinal region (Fig. 2*D*, navy). We repeated this computation with CAZyme abundances in place of genus abundances in order to quantify CAZyme variability across host species in each gastrointestinal region (Fig. 2*D*, green). We used bootstrapping across abundance vectors to compare variability values between pairs of regions (see *Materials and Methods* for details).

We see in Fig. 2*D* that values of FAVA for functional data (CAZyme, green) are about one-tenth those of taxonomic data (genus, navy), confirming that functional redundancy in the ruminant microbiome leads to much lower functional than taxonomic variability across host species. We established above that the compositional variability of genus abundances across all host individuals was lowest in the large intestine; by contrast, in Fig. 2*D*, *Inset*, the variability of CAZyme abundances is as low in the stomach (blue labels) as in the large intestine (red labels) (pairwise bootstrap comparisons between regions in the stomach and regions in the large intestine, one-sided *P* > 0.1 for each of 12 pairs). This comparison might suggest that there is more functional redundancy in the stomach than in the large intestine, in the sense that similar levels of CAZyme variability are obtained from a much greater taxonomic variability in the stomach.

In summary, through our analyses of ruminant microbiomes, FAVA allows us to capture the variability of high-dimensional data in a single number that can be easily compared across regions, species, or data types. The analysis finds that within each host species, both taxonomic and CAZyme community composition are most variable across host individuals in the small intestine.

### Defining Weighted FAVA.

**Fig. 3.**
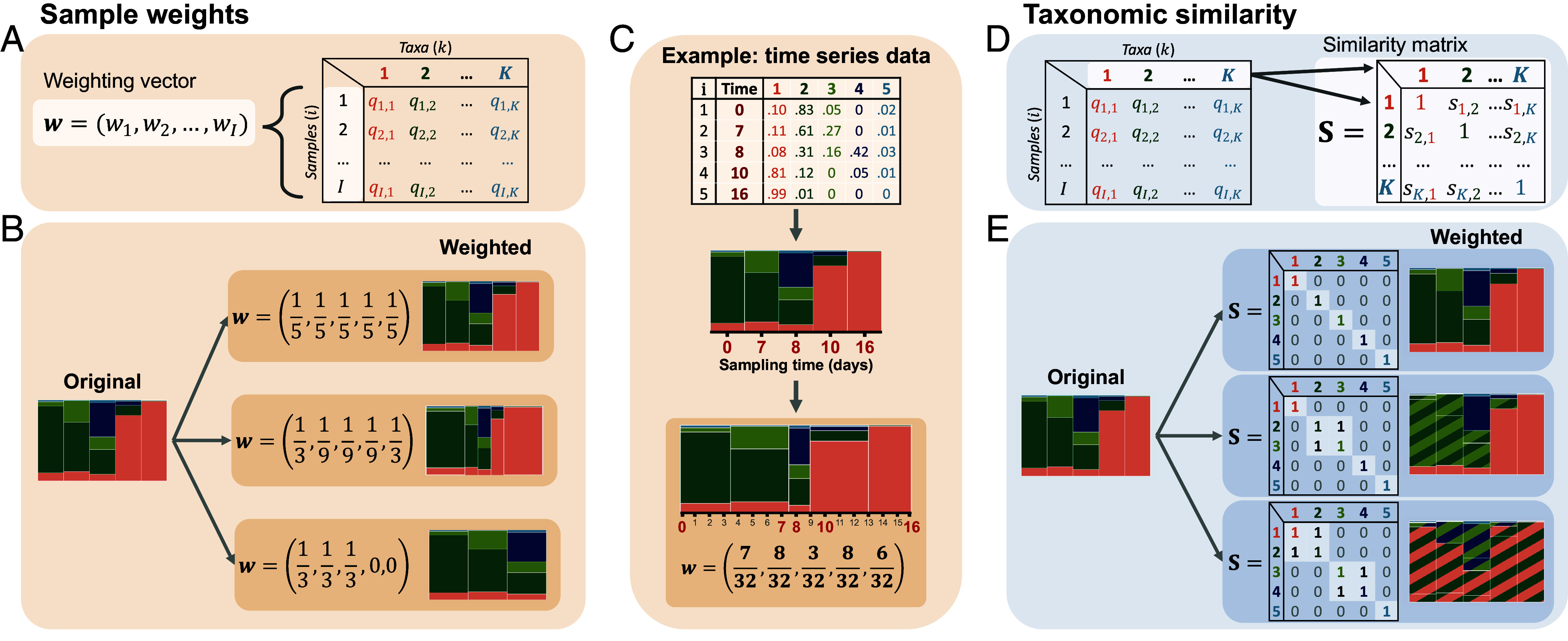
The FAVA framework can account for both uneven sample weights and information about the relatedness of taxa. (*A*) FAVA can be weighted by incorporating a normalized weighting vector (**w**). (*B*) Changing the weighting vector, **w**, changes the emphasis placed on each sample by FAVA. The bar plots on the *Right*-hand side represent how FAVA sees the original data under each weighting vector. (*C*) An OTU table with a column representing the collection time of each sample (*Top*) can be visualized as a stacked bar plot, with each bar corresponding to one sample (*Middle*). We can account for uneven sampling times by incorporating a weight vector **w** (*Bottom*) computed using Eqs. **5** and **6**. The *Bottom* bar plot represents how FAVA sees these data when this weight vector is used. (*D*) FAVA can also incorporate a similarity matrix (**S**) that represents the relatedness of each pair of taxa. Values can range between 0 and 1, equaling 0 if two taxa are unrelated and 1 if they are identical. (*E*) The coloring of the bar plots on the *Right*-hand side represents how FAVA sees the samples when they are weighted by each similarity matrix. Taxa 2 and 3 are treated as identical in the *Middle* example. In the *Bottom* example, taxa 1 and 2, and taxa 3 and 4 are considered identical. Although we use only zeroes and ones in this schematic, fractional values can be used to represent intermediate levels of similarity.

Our initial definition of FAVA (Eq. **4**) does not account for 1) differential weighting of rows (e.g., weighting based on time or distance between samples) or 2) similarities between columns (e.g., phylogenetic similarity between taxa). We now introduce a weighted version that allows for both uneven weighting of samples and for incorporation of information about the relatedness of taxonomic categories (Fig. 3).

First, sample weights are desirable when there is an uneven spatial or temporal distribution of samples, for example, if the experimental design includes some weekly samples and some daily samples. In this case, incorporating sample weights allows for greater emphasis on weekly samples, which inform the composition during a seven-day window, than on daily samples (e.g., Fig. 3*C*). Second, incorporating similarity among columns is valuable when the data include some taxa that are closely related and others that are more distant. This weighting helps make the measure more biologically informed, leading to higher weighted FAVA when the taxa that vary in abundance between samples are more distantly related.

We address row weights by incorporating into FAVA a weighting vector $w=(w_{1},w_{2},\ldots ,w_{I})$ that allows for varying emphasis of different samples (Fig. 3*A*–*C*). Each entry *w*_*i*_ determines the weight placed on sample *i* in the computation of weighted FAVA, and all *w*_*i*_ sum to 1. The default weighting vector assigns identical weight to each sample (Fig. 3*B*, *Top* example). Uneven weights change the emphasis on the different rows; those with larger values contribute more to the diversity calculation (Fig. 3*B*, *Middle* and *Bottom* example). When analyzing time series data, with each sample *i* corresponding to a time *t*_*i*_ between the start, *t*_1_, and the end, $t_{I},$ a natural choice is to weight each sample *i* by half the distance between the previous sampling time ($t_{i-1}$) and the subsequent sampling time ($t_{i+1}$) (Eq. **5**), normalized by the study duration ($T=t_{I}-t_{1}$) so that the weights sum to 1 (Eq. **6**). We provide an example of such weights derived from time series data in Fig. 3*C*.

We address similarities among columns by incorporating a similarity matrix, **S** (Fig. 3*D*). For each pair of taxa, this matrix contains a similarity scaled from 0 to 1. Entry $s_{k,\ell }$ of the similarity matrix **S** represents the similarity of taxa *k* and *ℓ*: $s_{k,\ell }=0$ if taxa *k* and *ℓ* are totally dissimilar, $s_{k,\ell }=1$ if taxa *k* and *ℓ* are identical, and intermediate values represent partial similarity. The diagonal elements of **S** all equal 1, because each taxon is identical to itself. The default similarity matrix is the identity matrix, which has zeroes for all off-diagonal elements (Fig. 3*E*, *Top* example). When ones are placed in off-diagonal elements of the matrix, the corresponding pair of taxa are treated as identical. For example, in the *Middle* example of Fig. 3*E*, taxa 2 and 3 are considered identical, as reflected in the coloring of taxa in the vertical bars to the *Right*. The similarity can be chosen to represent any relevant similarity concept, such as phylogenetic, genetic, or functional similarity.

We explain in *Materials and Methods* how we incorporate both *w*_*i*_ and **S** into equations for $\Delta_{S}$ and $\Delta_{T}$ (Eqs. **9** and **10**), resulting in an expression for weighted FAVA that considers both uneven row weights and nontrivial column similarities (Eq. **11**). Weighted FAVA (Eq. **11**) reduces to unweighted FAVA (Eq. **4**) when $w_{i}=\frac{1}{I}$ and $S=I_{K}$, a matrix with all *K* diagonal elements equal to 1 and all off-diagonal elements equal to 0 (*Top* examples of Fig. 3 *B* and *E*, respectively).

### Temporal Variability and Antibiotic Perturbation in the Human Gut Microbiome.

**Fig. 4.**
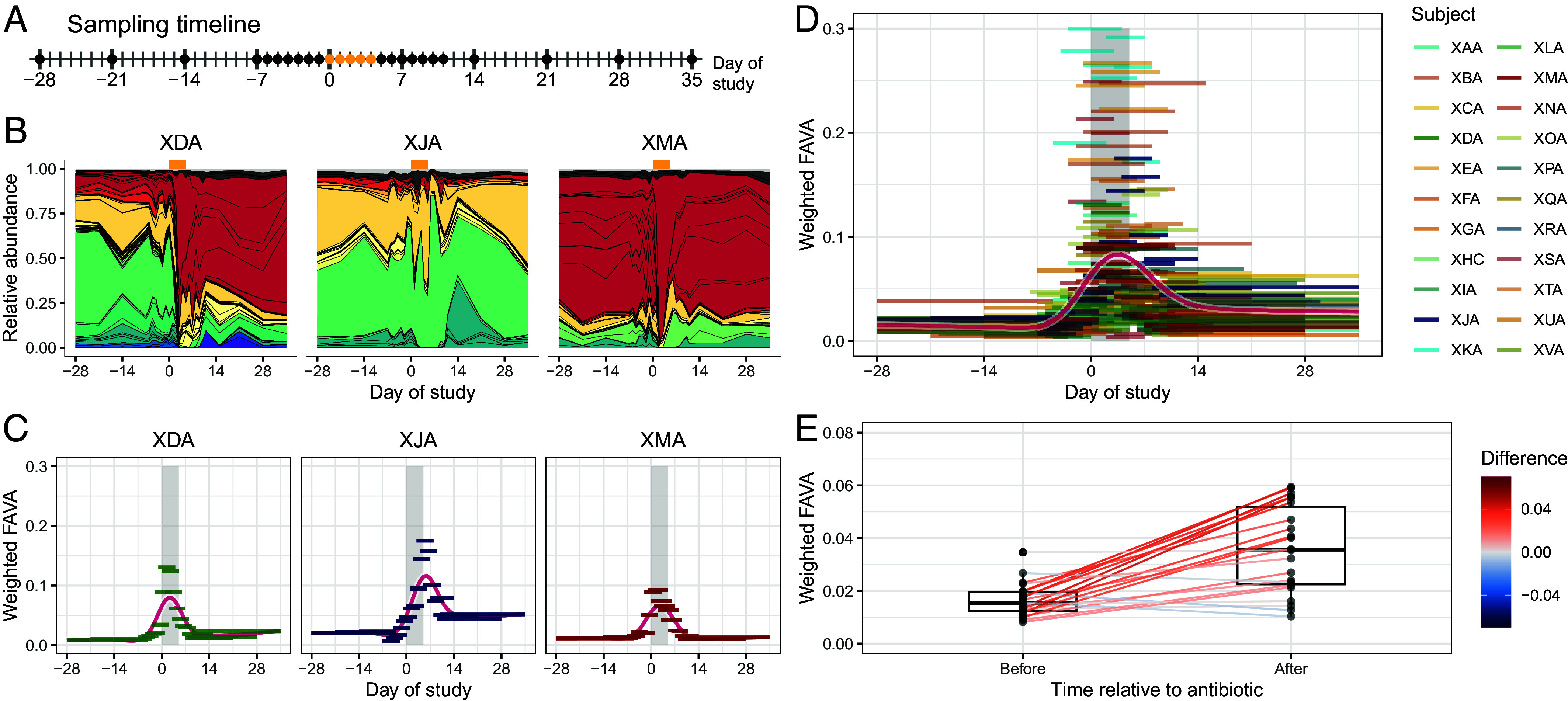
Weighted FAVA quantifies changes in the temporal variability of human gut microbiomes following an antibiotic perturbation. (*A*) Sampling timeline. Points correspond to sampling days. Samples are collected weekly for nine weeks, with daily sampling for three weeks beginning one week before antibiotics. Gold points denote days 0 to 4, during which the subjects took the antibiotic ciprofloxacin. (*B*) Selected relative abundance plots. Each three-letter code refers to one of 22 subjects. Abundances between sampling times are interpolated by drawing straight lines between abundances for adjacent time points. Colors denote bacterial families and black lines delineate bacterial species. All 22 subjects and the color legend for bacterial families are shown in *SI Appendix*, Fig. S2. (*C*) Sliding windows of weighted FAVA for selected subjects. For each subject, we generate sliding windows six samples wide (horizontal bars). The vertical position of each bar is determined by the value of weighted FAVA computed across the six samples in that window. The horizontal breadth of each bar encompasses the time during which the six samples were collected. The vertical gray bar denotes the time during which the subjects were taking antibiotics. The red curve is a smoothing spline fit to all data points with 12 degrees of freedom. Low values of weighted FAVA suggest community composition is stable in a window, whereas high values of weighted FAVA imply temporal variability. (*D*) Sliding windows of weighted FAVA for all subjects. We compute weighted FAVA in six-sample sliding windows for each of the 22 subjects. The red curve is a smoothing spline fit to all data points with 12 degrees of freedom. (*E*) Weighted FAVA increases after antibiotic perturbation. We compute weighted FAVA for each subject either before (days −28 to −1) or after antibiotic perturbation (days 5 to 35), excluding the period during which subjects were taking antibiotics. FAVA is weighted based on both phylogenetic similarity among bacterial species and time between samples. Lines connect values of weighted FAVA for the same subject before and after antibiotic perturbation. Lines are colored according to the difference in weighted FAVA (after minus before). Across all subjects, weighted FAVA increases significantly after antibiotic perturbation (Wilcoxon signed rank test, one-sided $P<10^{-4}$).

To demonstrate weighted FAVA as a measure of temporal microbiome variability, we apply it to data from a longitudinal study of gut microbiome composition after antibiotic perturbation (43). Among 48 subjects, we focused on 22 who took a course of the antibiotic ciprofloxacin midway through the study. For these subjects, stool samples were collected at 26 time points—weekly samples for nine weeks, as well as daily samples for the three weeks surrounding the antibiotic course (Fig. 4*A*). Xue et al. (43) inferred the relative abundances of bacterial species over time by shotgun metagenomic sequencing of each sample (Fig. 4*B*, for three of the 22 subjects). We use weighted FAVA to quantify both the impact of the antibiotic perturbation on temporal microbiome variability and the duration of this impact. To account for both the nonuniform sampling timeline and the broad taxonomic diversity of the sampled species, we weight FAVA by both the time intervals between stool samples and the phylogenetic similarity among species. We derive the phylogenetic similarity matrix from an established phylogenetic tree of bacterial species (44), as discussed in *Materials and Methods*.

We first explored how the temporal variability of the gut microbiome changes after an antibiotic perturbation. We find that weighted FAVA is significantly higher after the perturbation than before (Wilcoxon signed-rank test comparing postantibiotic to preantibiotic weighted FAVA values across 22 subjects, one-sided $P<10^{-4}$), suggesting that microbiome composition is more temporally variable after antibiotic perturbation (Fig. 4*E*). This result is robust to variability across subjects in the numbers of samples collected in the pre- and postantibiotic periods (*SI Appendix*, Fig. S3).

We explored temporal variability at smaller timescales by computing weighted FAVA in sliding windows across the study period. This increased granularity allowed us to quantify changes in temporal variability over the course of the study. Fig. 4*D* shows weighted FAVA in sliding windows six samples wide, with a median of 20 (overlapping) windows per subject. The sliding window analysis allows us to characterize the timeline of the antibiotic perturbation. We find that, for most subjects, the antibiotic perturbation results in a lasting increase in microbiome variability: Across the 30 d following completion of the antibiotic, only 8 of the 22 subjects returned to their initial variability level (Fig. 4*D*). However, high levels of variability tend to last for only one or two weeks postantibiotic: While all subjects began with sub-0.05 values of weighted FAVA and 18 of the 22 subjects exceeded 0.05 during the antibiotic period, 11 of these 18 subjects returned to weighted FAVA levels below 0.05 beginning one week postantibiotic, and 16 of these 18 had sub-0.05 weighted FAVA levels by their final sliding window.

Finally, our sliding window approach allows us to characterize temporal dynamics based on local temporal variability alone. For example, consider subjects XDA, XJA, and XMA, whose variability dynamics are highlighted in Fig. 4*C*. The microbiome of subject XJA does not stabilize postantibiotic, remaining highly variable through the end of the study period. Subjects XDA and XMA, on the other hand, both return to low variability levels within seven days of the conclusion of antibiotics. However, Fig. 4*B* reveals that these subjects represent two different responses to the antibiotic perturbation. Whereas XMA returns to the original compositional state after the antibiotic perturbation, XDA settles at a compositional state very different from the initial microbial community. This example highlights that, by computing (weighted) FAVA on small windows, we can identify periods of temporal stability in microbiome composition, even when the microbiome has stabilized at a compositional state different from its initial state.

### R Package.

We have implemented the FAVA framework in an R package, titled *FAVA*, which is available for download from CRAN, the standard public repository of R packages. Details on the installation and usage of *FAVA* are available on the package website, MaikeMorrison.github.io/FAVA. The package contains a function that can compute FAVA, weighted FAVA, and FAVA normalized by the upper bound given the abundance of the most abundant taxon. It also has functions to compute these three versions of FAVA in sliding windows and to visualize sliding window results in plots such as those in Fig. 4 *C* and *D*. The *FAVA* package can also visualize relative abundance data in stacked bar plots, and it can statistically compare groups of samples with bootstrapping. The *FAVA* R package is accompanied by a tutorial for its application to microbiome data. The tutorial is available at MaikeMorrison.github.io/FAVA/articles/microbiome_tutorial.html.

## Discussion

We have introduced an index to quantify variability across samples of microbiome composition. We defined the measure through an analogy with the population-genetic statistic *F*_*ST*_, considering microbiome samples in place of populations and microbial taxa in place of alleles. FAVA equals 0 if and only if all microbiome samples are identical, and 1 if and only if each sample contains only a single taxon and more than one taxon is present across all samples (Fig. 1). FAVA can be used as a measure of compositional variability across time points, spatial sampling locations, host individuals, or replicates, quantifying the temporal variability, spatial heterogeneity, or replicability of microbial communities. Because FAVA takes values between 0 and 1 irrespective of the number of sampled taxa, we can compare FAVA values between very different datasets, such as data on abundances of different taxonomic categories.

To demonstrate the FAVA framework’s performance as a measure of microbiome variability across many samples, we analyzed two microbiome datasets: an investigation of ruminant microbiome composition along the gastrointestinal tract (38), and a longitudinal study of human gut microbiome composition before and after an antibiotic perturbation (43). In the ruminant data, we found that compositional variability across individuals—either within a host species or across host species—was consistently lower at the end of the gastrointestinal tract than in the middle, supporting the view that substantial interindividual heterogeneity is missed when microbiomes are monitored by fecal sampling alone (Fig. 2 *B* and *D*) (18, 36). We found that, in all gastrointestinal regions, taxonomic abundances were much more variable across individuals than were functional abundances, a result that corroborates observations of microbial functional redundancy in the gastrointestinal tract (Fig. 2*D*) (39).

In the human microbiome data, we found that antibiotic perturbations destabilize microbial communities, resulting in elevated temporal variability following an antibiotic (Fig. 4*E*). Computing weighted FAVA in sliding windows across temporal samples for each subject increased the granularity of this analysis. Although elevated variability lasted for only one to two weeks postantibiotic on average, few subjects returned to preantibiotic variability levels during the study duration (Fig. 4 *C* and *D*). We also highlighted the FAVA framework’s ability to quantify temporal variability separate from compositional state by focusing on subjects XDA and XMA, who returned to their preantibiotic variability levels (Fig. 4*C*) even though only XMA returned to the original composition (*SI Appendix*, Fig. S4).

We introduced two extensions of FAVA: weighted FAVA (Eq. **11**), which can incorporate both similarity among taxa and distance between samples into the computation, and normalized FAVA, which accounts for the abundance of the most abundant taxon, allowing for more meaningful measurement of variability across small numbers of samples. In our analysis of human gut microbiome data over time (43), the use of weighted FAVA helped to account for both the combination of weekly and daily samples and the broad range of species appearing in the data.

FAVA values can be influenced by the choice of weights. For example, *SI Appendix*, Fig. S5 presents two hypothetical OTU tables with a large difference in FAVA when weighted by taxonomic similarity, despite having identical unweighted FAVA values. Nevertheless, in our analysis of human microbiome data, although individual FAVA values shift with the incorporation of weights, FAVA values computed across postantibiotic samples are consistently higher than those computed across preantibiotic samples, irrespective of weighting by sampling times, taxonomic similarity, or both (*SI Appendix*, Fig. S6).

Analyzing a higher taxonomic level can be viewed as a special case of weighting by taxonomic similarity. For example, to analyze family abundances in place of species abundances, we would define each entry $s_{k,\ell }$ of the species similarity matrix to equal 1 if species *k* and *ℓ* belong to the same family, and 0 otherwise. The taxonomic similarity matrix considered in *SI Appendix*, Fig. S5, for example, is equivalent to supposing that taxa *I* and *K* are each in separate families, whereas taxa *J* and *L* are in the same family. The result of this figure can consequently be interpreted to mean that matrix 1 has higher FAVA when computed using species (unweighted) rather than family abundances (weighted), while matrix 2 has lower FAVA when computed using species (unweighted) rather than family abundances (weighted).

We observe a similar composition-dependent relationship between taxonomic level and FAVA results in the data from Xue et al. (43) (*SI Appendix*, Fig. S7*A*). We computed FAVA across all samples from each antibiotic-taking subject from Xue et al. (43) using relative abundances of either bacterial families or species. Considering all subjects together, we do not observe a significant difference in FAVA values between the two levels of analysis (Wilcoxon signed rank test, *P* = 0.17). However, many individuals exhibit sizeable changes in FAVA values depending on the taxonomic level analyzed. *SI Appendix*, Fig. S7*B* highlights the compositions of the three subjects with the largest difference (XAA), smallest difference (XDA), and nearest difference to zero (XGA), comparing FAVA values computed using species and family abundances. Subject XAA’s higher species-level than family-level FAVA value is driven by large shifts in species composition within a single family whose abundance remains relatively constant, similar to matrix 1 in *SI Appendix*, Fig. S5. Subject XDA’s higher family-level than species-level FAVA value is a result of a large shift in abundances of families containing many component species, each with only small shifts in abundance—similar to matrix 2 in *SI Appendix*, Fig. S5. Finally, the species and family abundances in subject XGA follow very similar trajectories, producing similar species and family-level FAVA values.

We emphasize that comparisons of FAVA values between datasets with different numbers of categories, such as between species and family abundances (*SI Appendix*, Fig. S7), or between taxonomic and functional abundances (Fig. 2*D*), are enabled by the mathematical design of the FAVA measure. Under a Dirichlet model describing abundances in a set of categories, FAVA depends on the Dirichlet variance but does not otherwise depend on the abundances themselves; simulation of OTU tables in two scenarios, with 3 and 99 taxa, illustrates an identical, linear relationship with Dirichlet variances used for the simulations, irrespective of the number of taxa (*SI Appendix*, Fig. S1*A*). As an alternative to FAVA, the variability among a set of samples can also be measured with the mean of a pairwise statistic across all pairs of samples; in the same simulations of *SI Appendix*, Fig. S1*A*, computing one such statistic, the mean Bray–Curtis dissimilarity across pairs of samples, we observe in *SI Appendix*, Fig. S1*B* a strong dependence of the statistic on the number of taxa in the OTU table, so that it cannot be straightforwardly used to compare variability between tables with different numbers of categories.

We note that in the human microbiome analysis, we might have expected FAVA values to depend on data quality, as measured by the number of sequence reads used to estimate the relative abundances of bacterial taxa in microbiome samples. Variation in sequencing depth across samples could lead to varying accuracy in the estimation of abundances of bacterial taxa across samples, potentially shaping results of the FAVA framework. However, when subsampling reads from each microbiome sample and recomputing FAVA on the subsampled datasets, we find that FAVA values are largely unchanged, so that the sequencing depth is likely sufficient for their accurate estimation (*SI Appendix*, Fig. S8).

Our framework, which we have implemented in an R package, contributes to a large body of methods for the analysis of microbiome relative abundance data (30, 31). We emphasize, however, that the FAVA framework is a multisample compositional variability measure, setting it apart from the many existing measures of pairwise compositional similarity, such as Unifrac, Bray–Curtis dissimilarity, and the Jensen–Shannon divergence (Fig. 1*A*) (26, 27, 45). For example, two separate collections of microbiome samples can have identical values of FAVA, but wildly different mean compositions (e.g., Fig. 4 *B* and *C*). Similar results in the FAVA framework therefore reflect similarities in the spatial or temporal dynamics shaping variability, not compositional similarity. The FAVA framework complements diversity statistics such as the Gini-Simpson index, which summarize the diversity of taxa present in each sample rather than the variability of taxa across samples. For example, in the ruminant analysis, the Gini-Simpson diversity generally increases from the beginning to the end of the gastrointestinal tract, whereas FAVA peaks in the small intestine (*SI Appendix*, Fig. S9). The FAVA framework builds on a rich literature of frameworks for hierarchical partitioning of genetic, taxonomic, and phylogenetic diversity across individuals and communities (46–50); indeed, *F*_*ST*_ has sometimes been used as a measure of compositional variability in ecological contexts (51).

Future applications of the FAVA framework can span the range of questions that researchers pose about compositional variability, from understanding temporal variability in infant microbiomes (52, 53) to quantifying the repeatability of community assembly across experimental replicates to identifying the timing of compositional stability in serial passaging experiments (9, 12). Because the FAVA framework measures a fundamentally different phenomenon relative to existing methods for microbiome analysis, it can facilitate studies of previously challenging research questions relating to temporal stability, individual heterogeneity, spatial variability, and replicability.

## Materials and Methods

### Notation.

*Q* denotes an OTU table with *I* rows, each representing a microbiome sample, and *K* columns, each representing a microbial species, OTU, genus, functional unit, or other such category. Entry $q_{i,k}$ represents the relative abundance of taxon *k* in sample *i*. Each row must sum to 1. We use “sample *i*” to refer to row *i* of *Q*$\left(q_{i,*}\right)$.

### Bootstrapping Protocol.

We use bootstrapping (54) to determine whether two values of unweighted, weighted, or normalized FAVA are significantly different. Consider two OTU tables, *A* with *n* rows and *B* with *m* rows. The observed difference in (unweighted, weighted, or normalized) FAVA values between these two matrices is $D_{\text{obs}}=F_{\mathrm{ST}}\left(A,w,S\right)-F_{\mathrm{ST}}\left(B,w,S\right)$. Our null hypothesis is that there is no difference in (unweighted, weighted, or normalized) FAVA values between the communities sampled to form tables *A* and *B*.

To test this hypothesis, we first merge the two OTU tables into a single matrix, *Q*_null_, which has *n* + *m* samples corresponding to the samples in *A* and *B*. We then randomly draw *n* or *m* rows with replacement from *Q*_null_ to generate bootstrap replicates for *A* and *B*, *A*_boot_ and *B*_boot_ respectively. Finally, we compute the difference in (unweighted, weighted, or normalized) FAVA values between these bootstrap replicate matrices, $D_{\text{boot}}=F_{\mathrm{ST}}\left(A_{\text{boot}},w,S\right)-F_{\mathrm{ST}}\left(B_{\text{boot}},w,S\right).$ Repeating this procedure many times (e.g., 1,000 times) to generate many values of *D*_boot_ results in a bootstrap distribution of differences in (unweighted, weighted, or normalized) FAVA values between *A* and B.

We test our null hypothesis that there is no difference in (unweighted, weighted, or normalized) FAVA values between *A* and *B* by comparing the observed difference, *D*_obs_, to the bootstrap distribution of differences. We obtain a one-sided *P*-value by computing the proportion of bootstrapped differences *D*_boot_ that are either greater than or less than the observed difference *D*_obs_. We obtain a two-sided *P*-value by comparing $|D_{\text{boot}}|$ to $|D_{\text{obs}}|.$ A worked example of this computation is available in the *FAVA* R package vignette.

### Incorporating uneven sample weights.

For each sample i=1,2,…,I, we choose a weight $w_{i}\geq 0$ such that $\sum_{i=1}^{I}w_{i}=1.$ To evenly weight all samples, choose $w_{i}=\frac{1}{I}$ for all i. Uneven weights *w*_*i*_ can be chosen to account for properties such as sample size or the spatial or temporal distance between samples. If samples come from a time series, with *t*_*i*_ representing the sampling time of sample *i*, we recommend defining $w_{i}=\frac{d_{i}}{T}$ (Eq. **6**), where $T=t_{I}-t_{1}$ is the study duration and *d*_*i*_ is half the time from the sample before *i* to the sample after *i* (Eq. **5**):

[5] $$\begin{array}{c} d_{i}=\left\{\begin{array}{cc} \frac{t_{i+1}-t_{i-1}}{2}, & \text{if}2\leq i\leq I-1\\ \frac{t_{2}-t_{1}}{2}, & \text{if}i=1\\ \frac{t_{I}-t_{I-1}}{2}, & \text{if}i=I. \end{array}\right. \end{array}$$

Because $\sum_{i=1}^{I}d_{i}=T$,

[6] $$w_{i}=\frac{d_{i}}{T}$$

is a weight that sums to 1 over all *i* and represents the proportion of the study duration accounted for by sample *i*. Note that in the case of evenly spaced time samples, under the weighting $w_{i}=\frac{d_{i}}{T}$, the first and last sample are given half as much weight as the intermediate samples. This means that the uniform case is similar to but not exactly equal to the original, unweighted definition of *F*_*ST*_, which has $w_{i}=\frac{1}{I}.$

A standard definition for *F*_*ST*_ is $F_{\mathrm{ST}}=\left(\Delta_{T}-\Delta_{S}\right)/\Delta_{T}$ (Eq. **4**), where $\Delta_{S}$ is the mean sample Gini-Simpson diversity and $\Delta_{T}$ is the total Gini-Simpson diversity (Eqs. **2** and **3**):

$$\begin{array}{cc} \Delta_{S}\left(Q\right) & =1-\sum_{i=1}^{I}\frac{1}{I}\sum_{k=1}^{K}{\left(q_{i,k}\right)}^{2}\\ \Delta_{T}\left(Q\right) & =1-\sum_{k=1}^{K}{\left(\sum_{i=1}^{I}\frac{1}{I}q_{i,k}\right)}^{2}. \end{array}$$

We incorporate time information by replacing the uniform weights $\frac{1}{I}$ with not necessarily uniform weights $w=(w_{1},w_{2},\ldots ,w_{I})$:

$$\begin{array}{cc} \Delta_{S}\left(Q,w\right) & =1-\sum_{i=1}^{I}w_{i}\sum_{k=1}^{K}{\left(q_{i,k}\right)}^{2}\\ \Delta_{T}\left(Q,w\right) & =1-\sum_{k=1}^{K}{\left(\sum_{i=1}^{I}w_{i}q_{i,k}\right)}^{2}. \end{array}$$

$F_{\mathrm{ST}}\left(Q,w\right)=\left(\Delta_{T}\left(Q,w\right)-\Delta_{S}\left(Q,w\right)\right)/\Delta_{T}\left(Q,w\right)$ is thus a definition of *F*_*ST*_ that allows for uneven weighting of samples. Note that this weighting can account for differences in spacing between samples, but not for differences in relative ordering of samples.

#### Incorporating taxonomic similarity.

In addition to incorporating uneven sample weights, we may wish to account for the similarity between taxa. We capture information about the similarity among all *K* taxa through the symmetric, *K* × *K* similarity matrix **S**. The entry in row *k* and column *ℓ* of **S**, $s_{k,\ell }$, represents the similarity between taxon *k* and taxon *ℓ*. Diagonal elements satisfy $s_{k,k}=1$ because each taxon is identical to itself, and we define the similarity between identical taxa to be 1. Off-diagonal elements take values in [0,1], equaling 0 if two taxa are minimally similar, and 1 if they are identical. If **S** is the identity matrix (i.e., $s_{k,\ell }=0$ for all *k* ≠ *ℓ*), then all distinct taxa are treated as minimally similar and our weighted version of *F*_*ST*_ must reduce to its original, unweighted definition (Eq. **4**).

In order to incorporate **S** into the definition of *F*_*ST*_, we first introduce Leinster and Cobbold’s (55) idea of “mean ordinariness” across taxa in a microbiome sample. The “ordinariness” of taxon *k* in sample *i* is the mean similarity between that taxon and every other taxon in the sample, weighted by the taxon abundances. It is computed for each taxon by multiplying the similarity matrix (**S**) by the vector for sample *i* ($q_{i,*}$). This computation produces a vector whose $k^{\mathrm{th}}$ entry, ${\tilde{q}}_{i,k}$, represents the mean similarity between species *k* and a random taxon from sample *i*:

$$\begin{array}{cc} {\tilde{q}}_{i,k}={\left(Sq_{i,*}^{T}\right)}_{k} & =\sum_{\ell =1}^{K}s_{k,\ell }q_{i,\ell }\\ & =q_{i,k}+\sum_{\begin{array}{c} \ell =1\\ \ell \neq k \end{array}}^{K}s_{k,\ell }q_{i,\ell }\\ & \geq q_{i,k}. \end{array}$$

In other words, ${\tilde{q}}_{i,k}$ measures the ordinariness of taxon *k* within sample *i*. On one extreme,

$${\tilde{q}}_{i,k}=\sum_{\ell =1}^{K}1\cdot q_{i,\ell }=1$$

if taxon *k* is identical to all other taxa in sample *i*. In this case, taxon *k* is maximally ordinary in relation to the other taxa in the sample. On the other extreme,

$${\tilde{q}}_{i,k}=1\cdot q_{i,k}+\sum_{\begin{array}{c} \ell =1\\ \ell \neq k \end{array}}^{K}0\cdot q_{i,\ell }=q_{i,k}$$

if taxon *k* has similarity 0 to all other taxa in sample *i*. In this case, taxon *k* is minimally ordinary in relation to the other sampled taxa. The mean taxon ordinariness across all taxa in sample *i*, weighted by their abundances, is

[7] $$\sum_{k=1}^{K}q_{i,k}\cdot {\tilde{q}}_{i,k}.$$

This quantity has been explored in previous work on ecological diversity indices (55). It is large (i.e., approaching or equal to 1) if the sample is concentrated in a few very similar taxa, whereas it is small (i.e., approaching 0) if the sample is spread across many unrelated taxa. If **S** is the identity matrix, with ones along the diagonal and zeroes for off-diagonal elements, then ${\tilde{q}}_{i,k}=q_{i,k}$ for all *k* and Eq. **7** reduces to the mean taxon abundance across all taxa in sample *i*, $\sum_{k=1}^{K}{\left(q_{i,k}\right)}^{2}.$

We proceed by extending this idea of mean ordinariness into the framework of *F*_*ST*_. First, recall the original definition of the Gini-Simpson index (Eq. **1**), which can be interpreted as one minus the mean taxon abundance across all taxa in a sample:

$$\Delta \left(q_{i,*}\right)=\Delta \left(q_{i,1},q_{i,2},\ldots ,q_{i,K}\right)=1-\sum_{k=1}^{K}q_{i,k}\cdot q_{i,k}.$$

We incorporate the similarity matrix **S** into the Gini-Simpson index by replacing the mean abundance across taxa, $\sum_{k=1}^{K}q_{i,k}^{2}$, with the mean ordinariness across taxa, $\sum_{k=1}^{K}q_{i,k}\cdot {\tilde{q}}_{i,k},$ giving the following definition:

[8] $$\begin{array}{c} \begin{array}{cc} \Delta (q_{i,*},S) & =1-\sum_{k=1}^{K}q_{i,k}\cdot {\tilde{q}}_{i,k}\\ & =1-\sum_{k=1}^{K}q_{i,k}\cdot {\left(Sq_{i,*}^{T}\right)}_{k}. \end{array} \end{array}$$

Eq. **8** reduces to Eq. **1** if **S** is the identity matrix. In this case, each taxon is considered extraordinary, with similarity 0 to all other taxa. However, if **S** has nonzero off-diagonal elements, Eq. **8** is able to account for the similarity among taxa in its computation of diversity.

Finally, we extend Eq. **8** to define versions of $\Delta_{S}$ and $\Delta_{T}$:

$$\begin{array}{cc} \Delta_{S}\left(Q,S\right) & =1-\frac{1}{I}\sum_{i=1}^{I}\sum_{k=1}^{K}q_{i,k}\cdot {\left(Sq_{i,*}^{T}\right)}_{k}\\ \Delta_{T}\left(Q,S\right) & =1-\sum_{k=1}^{K}(\frac{1}{I}\sum_{i=1}^{I}q_{i,k})\cdot (\frac{1}{I}\sum_{i=1}^{I}{\left(Sq_{i,*}^{T}\right)}_{k}). \end{array}$$

Using these extensions of $\Delta_{S}$ and $\Delta_{T}$ in a computation of *F*_*ST*_ yields a compositional variability measure that accounts for taxonomic similarity: $F_{\mathrm{ST}}\left(Q,S\right)=\left(\Delta_{T}\left(Q,S\right)-\Delta_{S}\left(Q,S\right)\right)/\Delta_{T}\left(Q,S\right)$.

#### Simultaneously incorporating uneven row weights and taxonomic similarity.

We simultaneously incorporate both **S** and **w** into equations for $\Delta_{S}$ and $\Delta_{T}$ in order to develop a compositional variability statistic that accounts for both weighting of samples and similarity among taxa (Eqs. **9**–**11**):

[9] $$\Delta_{S}\left(Q,w,S\right)=1-\sum_{i=1}^{I}w_{i}\sum_{k=1}^{K}q_{i,k}\cdot {\left(Sq_{i,*}^{T}\right)}_{k}$$

[10] $$\Delta_{T}\left(Q,w,S\right)=1-\sum_{k=1}^{K}(\sum_{i=1}^{I}w_{i}q_{i,k})\cdot (\sum_{i=1}^{I}w_{i}{\left(Sq_{i,\cdot }\right)}_{k})$$

[11] $$F_{\mathrm{ST}}\left(Q,w,S\right)=\frac{\Delta_{T}\left(Q,w,S\right)-\Delta_{S}\left(Q,w,S\right)}{\Delta_{T}\left(Q,w,S\right)}.$$

If $w_{i}=1/I$ for all *i*, and **S** is the identity matrix, this weighted definition of *F*_*ST*_ (Eq. **11**) reduces to the unweighted version of *F*_*ST*_ (Eq. **4**).

### Computing the Phylogenetic Similarity Matrix.

In our analysis of human microbiome data (Fig. 4), we chose to weight FAVA by the phylogenetic similarity among the sampled bacterial species. We computed the phylogenetic similarity matrix through a two-step process. First, we computed the patristic distance between each pair of sampled bacterial species based on a microbial phylogeny from Nayfach et al. (44). We performed this computation with the “cophenetic.phylo” function in the *ape* R package (56). Second, we transformed the pairwise patristic distances, which range from 0 for identical species to ∼3.7 for very distantly related species, to similarities, which range from 0 for very distantly related species to 1 for identical species. We chose to convert the patristic distance between species *k* and *ℓ* ($d_{k,\ell }$) to a similarity ($s_{k,\ell }$) using the exponential transformation $s_{k,\ell }=\exp\left(-d_{k,\ell }\right)$.

Different transformations of distances to similarities result in different distributions of similarity values. In our case, the similarity matrix computed with the exponential transformation had a median value of 0.087, with first and third quartiles [0.067,0.119]. Other transformations are defensible as well. The linear difference transformation ($s_{k,\ell }=1-d_{k,\ell }/\max d_{k,\ell }$), for example, yields a mean value of 0.716, with first and third quartiles [0.685,0.752]. We note that the main results of Fig. 4 do not depend on the choice of transformation. In particular, irrespective of the transformation used, weighted FAVA values increase during the antibiotic perturbation and are significantly higher postantibiotic than preantibiotic (*SI Appendix*, Fig. S10).

### Datasets.

#### Ruminant data.

In our first data example, we analyzed genus and CAZyme abundances inferred from metagenomic sequencing of samples collected at 10 gastrointestinal regions from 37 ruminant host individuals representing 7 host species. This dataset was collected and published by Xie et al. (38). We downloaded the data from http://rummeta.njau.edu.cn/rumment/resource/metagenomicsPage. The genus abundances were found in the file “RGMGC.genus.profile.txt” which was available for download under the heading “Genus profile (genus abundance profile table for 370 GIT samples).” The CAZyme abundances were found in the file “RGMGC.cazy.profile.family.txt,” which was available for download under the heading “Cazy profile (Cazy abundance profile table for 370 GIT samples).” For both genera and CAZymes, the published data contained absolute abundances. We converted absolute abundances to relative abundances before performing our analyses.

#### Human microbiome data.

In our second data example, we analyzed data generated by Xue et al. (43).

## Supplementary Material

Appendix 01 (PDF)

## Data Availability

Previously published data were used for this work (38, 43).
