# Supplementary material for: Quantifying compositional variability in microbial communities with FAVA
Source: Proc Natl Acad Sci U S A. 2025 Mar 10;122(11):e2413211122. doi: 10.1073/pnas.2413211122 (PMC11929398; doi:10.1073/pnas.2413211122)
Supplement: Supplementary file 1 — Appendix 01 (PDF) [file pnas.2413211122.sapp.pdf]

Supporting Information for

Quantifying compositional variability in microbial communities with FAVA

Maike L. Morrison, Katherine S. Xue, Noah A. Rosenberg

Maike Morrison.
E-mail: maikem@stanford.edu

This PDF file includes:

Figs. S1 to S10
SI References

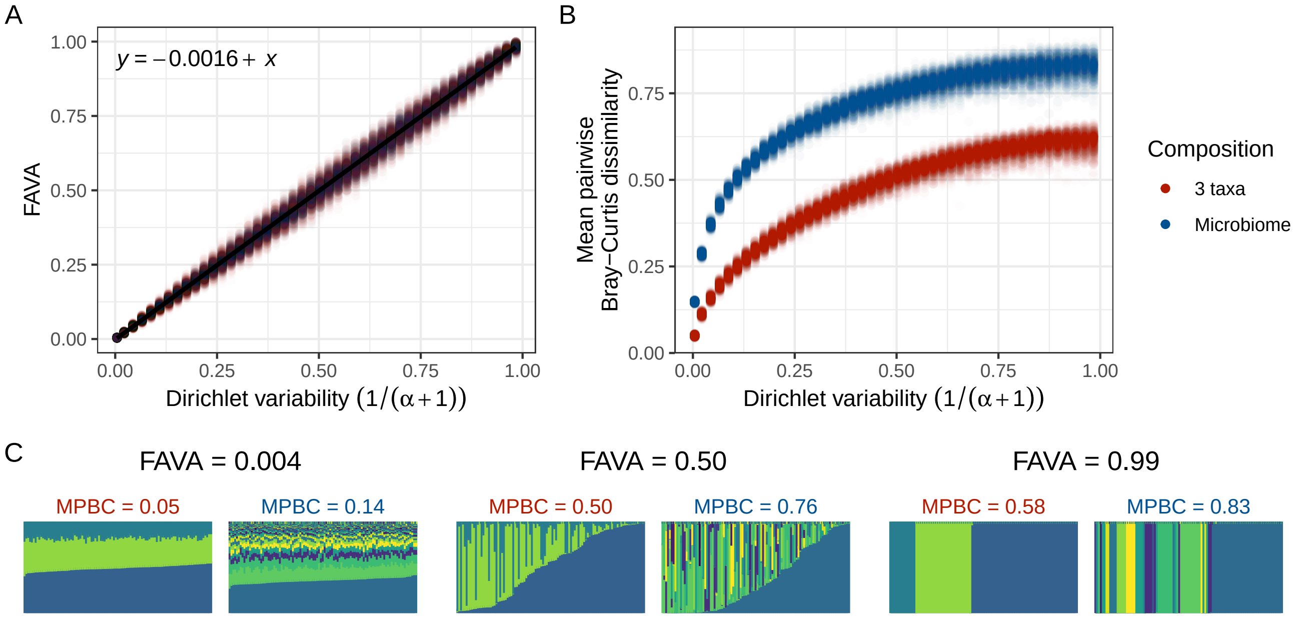

Fig. S1. FAVA, unlike mean pairwise Bray-Curtis dissimilarity, has a linear relationship with $1/(\alpha+1)$, a constant that scales the variances of abundances in a Dirichlet probability model, and does not depend on the number of taxa in a community. We used a Dirichlet model, $(\alpha\lambda)$, to simulate the relative abundances of taxa in a sample. The vector of means, λ, gives the mean relative abundances of the taxa: $\lambda_i = \mathbb{E}[q_{i,k}]$, where $q_{i,k}$ is the abundance of taxon i in sample k. For each simulation, we used one of two λ vectors: the "3 taxa" composition $\lambda = (\frac{1}{2}, \frac{1}{3}, \frac{1}{6})$ (red), or the 99-taxa "microbiome composition" (blue), whose λ follows the abundances of the 99 taxa present in the first microbiome sample collected from subject XAA in the data of (1): $\lambda = (0.35, 0.15, 0.10, 0.07, 0.06, 0.04, \ldots, 7.1\times10^{-6}, 4.3\times10^{-6}, 3.4\times10^{-6}, 2.4\times10^{-6})$. The variability parameter α sets the variance of each taxon abundance: $[q_{i,k}] = \lambda_i(1-\lambda_i)/(\alpha+1)$. For both compositions, we considered 46 values of α selected based on the microbiome composition to generate a range of Dirichlet variances for the most abundant microbiome taxon, $[q_1]$, given the mean abundance of the most abundant taxon was $\lambda_1 = 0.35$: $[q_1] = \lambda_1(1-\lambda_1)/(\alpha+1) = 0.001, 0.005, 0.010, 0.015, \ldots, 0.220, 0.225$; the largest value approaches the maximum variance, $\lambda_i(1-\lambda_i)/(0+1) = 0.228$. We simulated each OTU table (e.g., the six tables plotted in panel C) by drawing 100 samples from one of these Dirichlet distributions. We simulated 500 OTU tables for each combination of λ (3-taxa or microbiome) and α. **(A)** Relationship between FAVA and the proportionality constant for the Dirichlet variance, $1/(\alpha+1)$. The expected value of FAVA is equal to this constant, irrespective of the mean composition of the community (2). The ordinary least-squares regression line has slope 0.999 ($P < 10^{-16}$), intercept -0.0016 ($P < 10^{-15}$), and adjusted $R^2 = 0.994$. **(B)** Relationship between the mean pairwise Bray-Curtis dissimilarity (MPBC) and $1/(\alpha+1)$. The value of MPBC is sensitive to differences in the composition and has a nonlinear relationship with $1/(\alpha+1)$. **(C)** Pairs of OTU tables with low (FAVA $= 0.004$), medium (FAVA $= 0.50$), and high (FAVA $= 0.99$) compositional variability, but different mean composition (3 taxa or microbiome). Each pair of matrices has similar Dirichlet variability values (from left to right, values of $1/(\alpha+1)$ are: 0.004, 0.004; 0.459, 0.524; and 0.983, 0.983) but quite different MPBC values (red for 3 taxa composition, blue for microbiome composition). These matrices exemplify the pattern in panels A and B that FAVA reflects variance of composition across samples, but MPBC also reflects compositional differences.

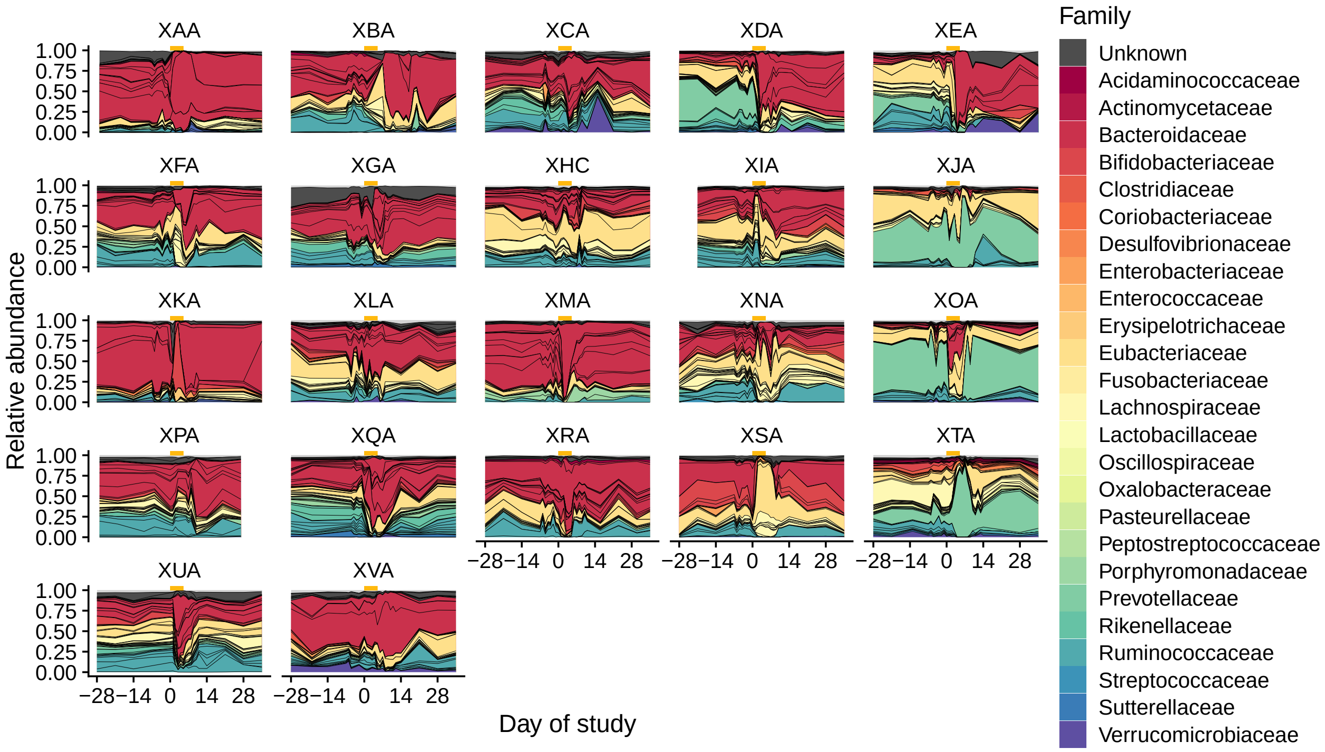

Fig. S2. Relative abundances of bacterial taxa over time for 22 subjects. Each three-letter code refers to one subject. Gold bars denote days 0-4, during which the subjects took the antibiotic ciprofloxacin. Samples are collected once weekly for 9 weeks, with daily sampling for three weeks beginning one week before antibiotics. Abundances between sampling times are interpolated by drawing straight lines between abundances for adjacent time points. Colors denote bacterial families and black lines delineate bacterial species. Species are identified by mapping metagenomic sequencing reads to a database of single-copy bacterial genes. The figure is adapted from Xue et al. (1).

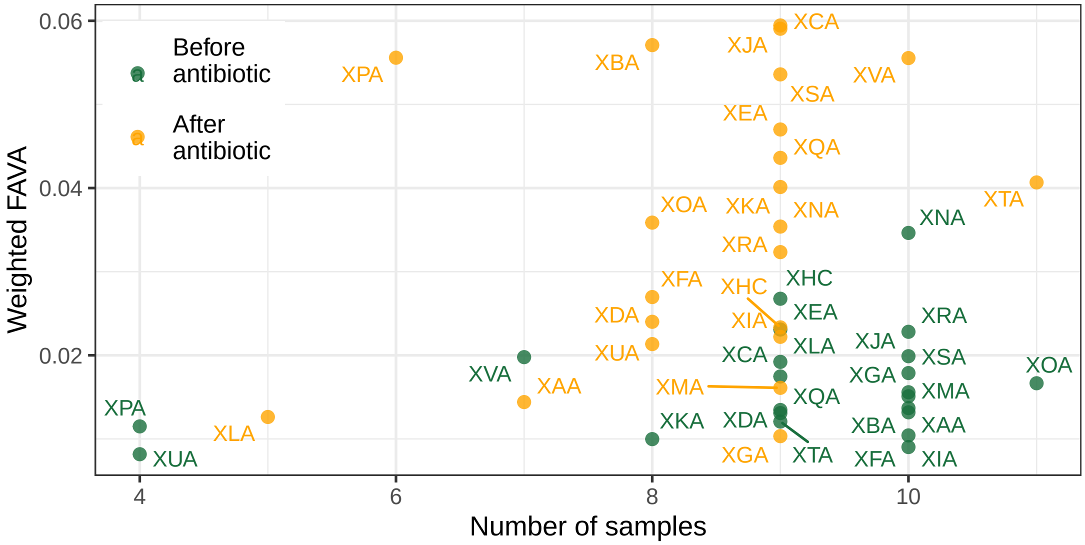

Fig. S3. Change in weighted FAVA before and after the antibiotic perturbation is driven by antibiotic, not by the number of samples before or after antibiotic. Each point represents an analysis of all samples collected from one of 22 subjects either before the antibiotic perturbation (green points; samples taken between day -28 and day -1) or after the antibiotic perturbation (orange points; samples taken between day 5 and day 35). The x-axis gives the total number of samples analyzed for each point, and the y-axis gives the value of weighted FAVA computed across these samples. The y-axis presents the same data shown in Figure 4E. While subjects tended to have more samples before than after the antibiotic perturbation (Wilcoxon signed rank test, one-sided $P = 0.013$), there was no correlation between weighted FAVA and the number of samples included in the computation (Spearman correlation $\rho = -0.001$, $P = 0.992$). We therefore conclude that the difference in weighted FAVA values observed in Figure 4E is driven by the antibiotic perturbation, not by differences in the numbers of samples included in computations of weighted FAVA.

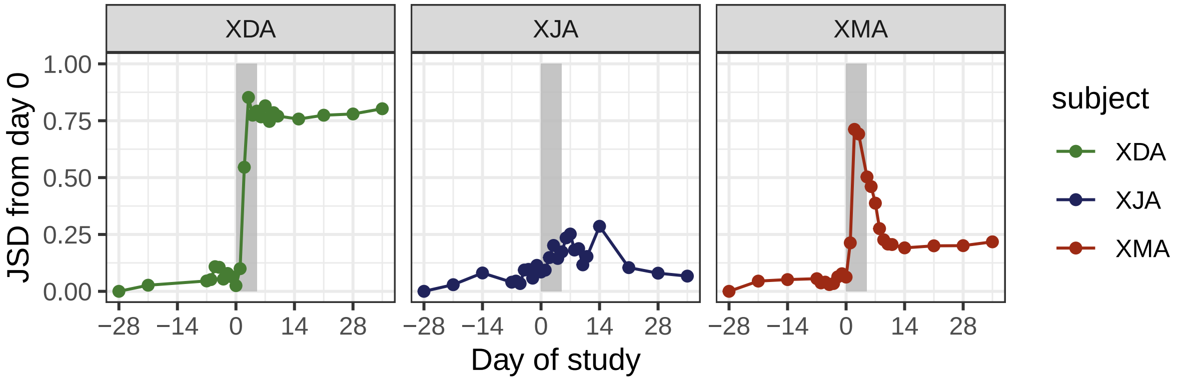

Fig. S4. The Jensen-Shannon divergence (JSD) from the initial timepoint captures compositional changes in microbial communities following antibiotic perturbation (grey bar). JSD is computed using species relative abundances.

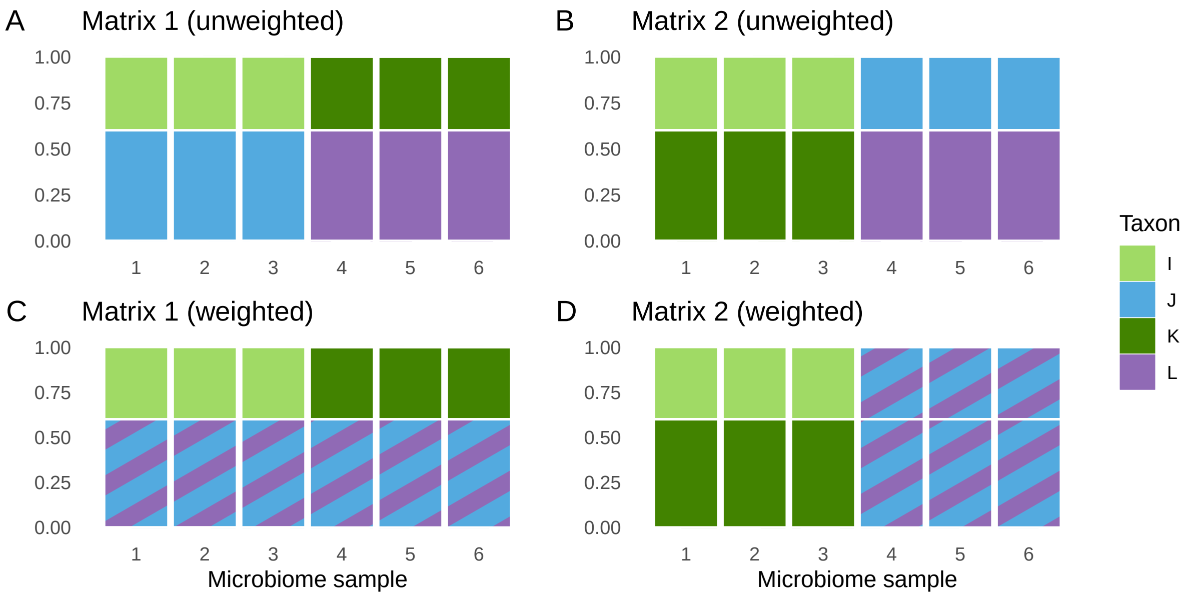

Fig. S5. The effect of incorporating taxonomic similarity into the FAVA computation depends on the taxonomic composition of the sampled communities. Each panel shows one matrix representing the compositions of six hypothetical microbiome samples. Without accounting for taxonomic similarity, both Matrix 1 (A) and Matrix 2 (B) have FAVA values of 0.35. We consider a similarity matrix that treats taxon J and taxon L as identical (similarity equal to 1), and all other distinct taxa as totally unrelated (similarity equal to 0). Incorporating this similarity matrix in the computation of FAVA decreases Matrix 1's FAVA value to 0.14 (C) and increases Matrix 2's FAVA value to 0.61 (D).

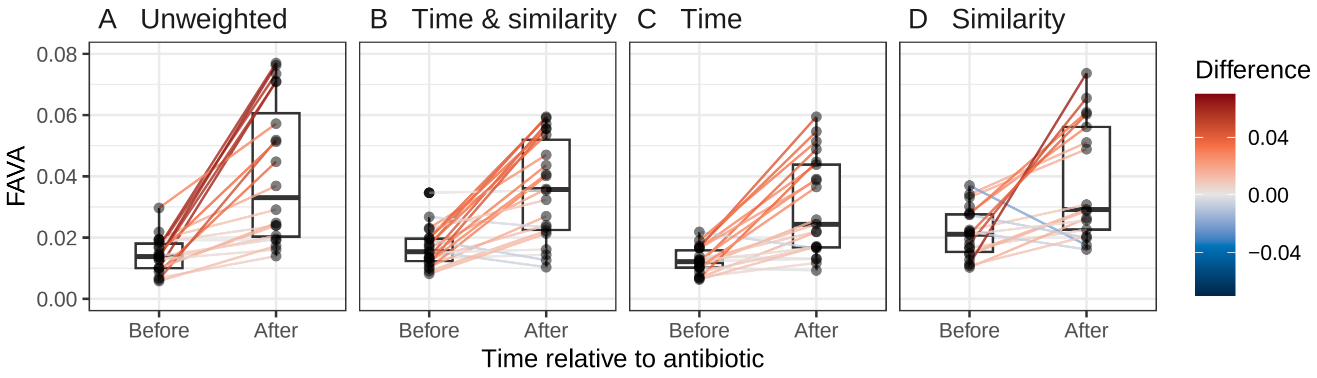

Fig. S6. Values in the FAVA framework increase after antibiotic perturbation irrespective of FAVA weighting. We compute weighted or unweighted FAVA for each subject either before the antibiotic perturbation (samples in days -28 to -1) or after the antibiotic perturbation (samples in days 5 to 35). Lines connect values of each FAVA category for the same subject before and after the antibiotic. The four panels correspond to four different types of weighting for the FAVA measure: **(A)** No weights. **(B)** Weighting by both time between samples and phylogenetic similarity among taxa. **(C)** Weighting just by time. **(D)** Weighting just by phylogenetic similarity. In all four panels, values are significantly higher after the antibiotic perturbation than before (Wilcoxon signed rank test, one-sided $P < 10^{-4}$ for each panel).

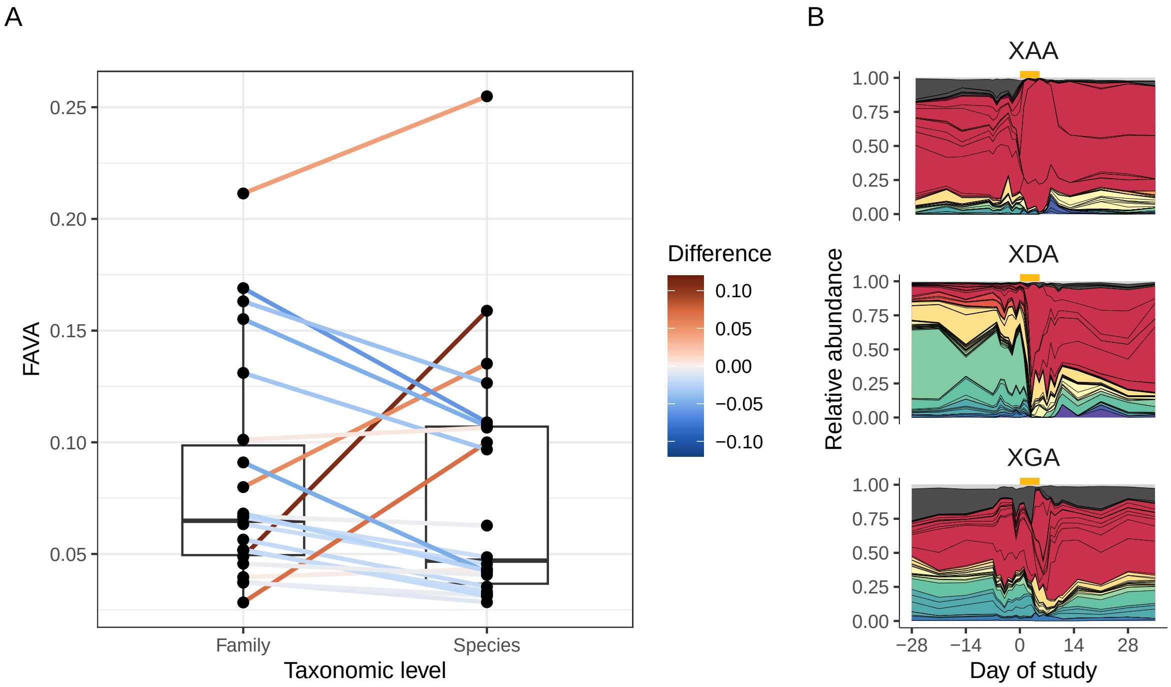

Fig. S7. Although the distributions of FAVA values computed on species and family abundances do not significantly differ, individual FAVA values can shift substantially with the taxonomic level analyzed. (A) FAVA computed on species or family abundances across all samples for each of the 22 subjects from Xue et al. (1). Lines connect analyses from the same subject and are colored according to the difference in FAVA values (species minus family). The two distributions do not significantly differ (Wilcoxon signed rank test, $P = 0.17$); 16 subjects have higher family than species-level FAVA values, 6 have lower family-level values. **(B)** Relative abundance plots for three highlighted subjects, following the style of Figure 4B: XAA with the maximum difference of 0.11, XDA with the minimum difference of -0.06, and XGA with the difference closest to 0, 0.004. XAA's large positive difference is driven by large shifts in species abundances within the same family. Conversely, XDA's large negative difference is due to a large shift in the abundance of a family abundance that contains many distinct species. Because XGA's species and family compositions generally move in parallel, species and family-level FAVA values are quite similar.

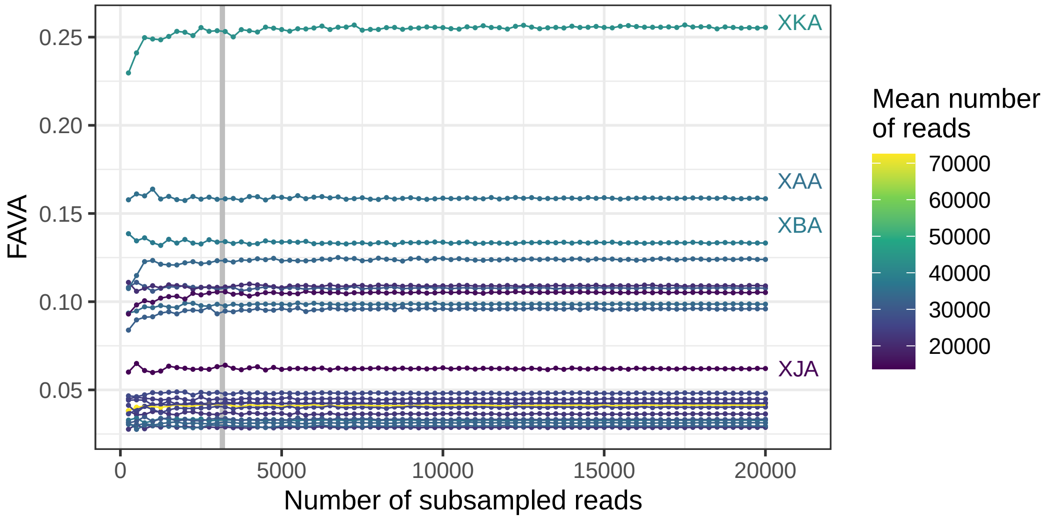

Fig. S8. FAVA values have little dependence on the number of reads in microbiome samples. The abundances we analyzed for human gut microbiomes were obtained by Xue et al. (1) by mapping shotgun metagenomic sequencing reads to a database of core, single-copy bacterial genes using MIDAS (3). To explore the influence of read depth on FAVA results, for each microbiome sample, we randomly subsampled the set of metagenomic reads that mapped to core genes down to a threshold (x-axis) less than or equal to the total number of mapped reads in the original microbiome sample. We performed this subsampling independently for each microbiome sample (i.e., each time point in one subject). If the threshold was greater than the number of reads mapping to core genes in the sample, then the full sample was used. We independently subsampled each microbiome sample to thresholds from 250 to 20,000 reads mapping to core genes, at intervals of 250 reads. We used the set of subsampled reads mapping to core genes from each microbiome sample to compute the corresponding species relative abundances in that microbiome sample. Finally, we computed FAVA using the subsampled microbiome species abundances, computing FAVA across all microbiome samples (timepoints) from a subject. The x-axis gives the subsampling threshold, and the y-axis gives the value of FAVA computed across all subsampled microbiome samples for each subject. The vertical gray line gives the threshold of reads mapping to core genes that was used for quality control ($10^{3.5}$). All samples with fewer than $10^{3.5}$ reads mapping to core genes were excluded from the original analyses. The color of a line denotes the mean number of reads that mapped to core genes across the microbiome samples for a subject.

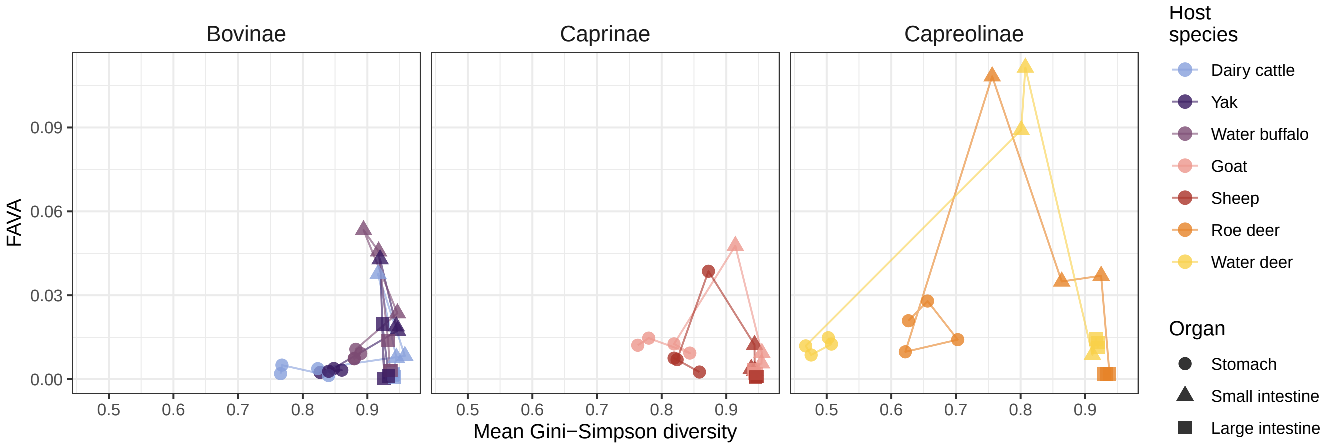

Fig. S9. FAVA across ruminant gastrointestinal samples is not correlated with mean Gini-Simpson diversity of the samples. Dots represent one gastrointestinal region in one host species. The x-axis gives the mean Gini-Simpson diversity across samples in that host and region; the y-axis gives the value of FAVA computed across these samples. Lines connect consecutive gastrointestinal regions in the same host species. Samples from the stomach (28 data points for 7 species and 4 regions) generally have lower mean Gini-Simpson diversity than samples from the small intestine (21 data points, Wilcoxon rank-sum test, $P < 10^{-6}$) or the large intestine (21 data points, Wilcoxon rank-sum test, $P < 10^{-13}$). The large intestine and small intestine do not have significantly different Gini-Simpson diversity (Wilcoxon rank-sum test, $P = 0.42$).

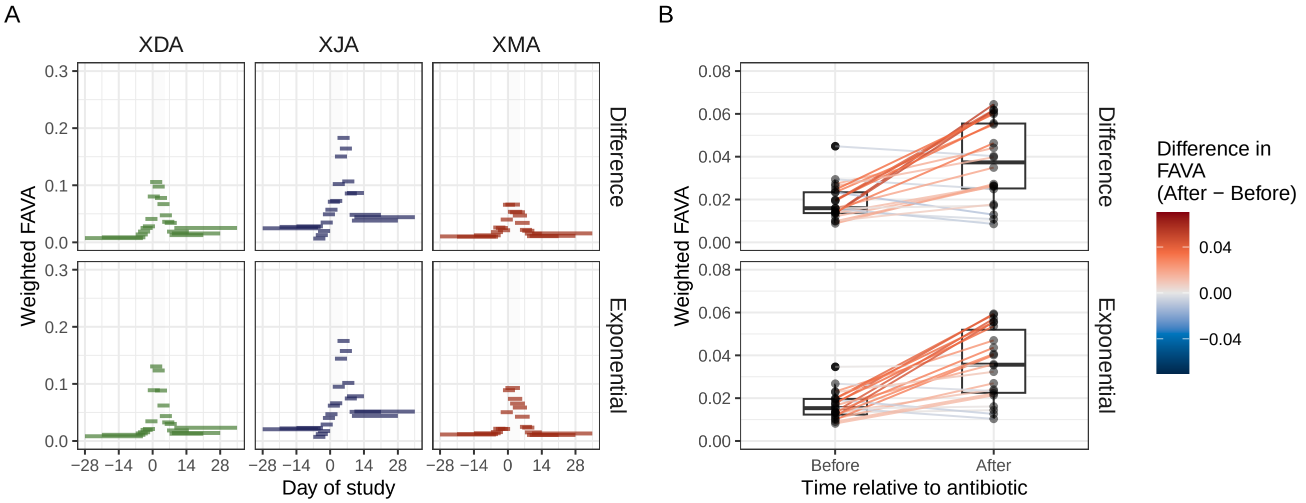

Fig. S10. Comparing results from Figure 4 when using different similarity matrices. Panel A reproduces Figure 4C; panel B reproduces Figure 4E. The top row of each panel gives the results using a similarity matrix defined from the patristic distance matrix using the linear difference transformation. The bottom row of each panel gives the results using the exponential transformation, which are the results presented in the main text. The results are largely consistent between the two transformations. For example, as is true for the exponential results presented in the main text, weighted FAVA values computed with the similarity matrix generated using the linear difference transformation are higher after antibiotic perturbation than before (Wilcoxon signed-rank test comparing post-antibiotic to pre-antibiotic weighted FAVA values across subjects, $P = 0.0003$).

References

1. KS Xue, et al., Prolonged delays in human microbiota transmission after a controlled antibiotic perturbation. *bioRxiv* p. 2023.09.26.559480 (2023).
2. ML Morrison, N Alcala, NA Rosenberg, FSTruct: An FST-based tool for measuring ancestry variation in inference of population structure. *Mol. Ecol. Resour.* **22**, 2614–2626 (2022).
3. S Nayfach, B Rodriguez-Mueller, N Garud, KS Pollard, An integrated metagenomics pipeline for strain profiling reveals novel patterns of bacterial transmission and biogeography. *Genome Res.* **26**, 1612–1625 (2016).
